# Waiting times in healthcare: equal treatment for equal need?

**DOI:** 10.1186/s12939-022-01799-x

**Published:** 2022-12-20

**Authors:** Juan David García-Corchero, Dolores Jiménez-Rubio

**Affiliations:** Department of Applied Economics, University of Granada, Granada, Spain

**Keywords:** Waiting times, Socioeconomic status, Inequalities, Primary care, Specialist care, National Health System, I11, I18

## Abstract

**Background:**

In many universal health systems, waiting times act as a non-monetary rationing mechanism, one that should be based on clinical need rather than the ability to pay. However, there is growing evidence that among patients with similar levels of need, waiting times often differ according to socioeconomic status. The mechanisms underlying inequality in access remain unclear.

**Methods:**

Using data for Spain, we study whether waiting times for primary and specialist care depend on patients’ socioeconomic status (SES). Additionally, we make use of the continuous nature of our data to explore whether the SES-related differences in waiting times found for specialist consultations vary among different points of the waiting time distribution.

**Results:**

Our results reveal the presence of a SES gradient in waiting times for specialist services explained on the basis of education, employment status and income. In addition, for primary care, we found evidence of a slightly more moderate SES gradient mostly based on employment status. Furthermore, although quantile regression estimates indicated the presence of a SES gradient within the distribution of waiting times for specialist visits, the SES differences attenuated in the context of longer waiting times in the public sector but did not disappear.

**Conclusion:**

Our findings suggest the principle of equal treatment for equal need, assumed to be inherent to national health systems such as the Spanish system, is not applied in practice. Determining the mechanism(s) underlying this selective barrier to healthcare is of crucial importance for policymakers, especially in the current COVID-19 health and economic crises, which could exacerbate these inequalities as increasing numbers of treatments are having to be postponed.

**Supplementary Information:**

The online version contains supplementary material available at 10.1186/s12939-022-01799-x.

## Introduction

The issue of waiting times for healthcare is a major topic of political discussion in most countries that enjoy a universal healthcare system [1]. Due to increasing pressures on health systems worldwide, treatments often have to be postponed, causing patients further deterioration in their health status. Moreover, long waiting times may be seen as a barrier to accessing health services, a question that is particularly sensitive for publicly funded health systems. When patients are dissatisfied with the health service, they may make insufficient use of it, with negative consequences for health levels in the population. Moreover, when waiting times are long, patients may opt out of the public health system, purchasing private health insurance or increasing out-of-pocket expenses. Privately insured individuals are less likely to favour increased spending on the NHS, or to view public healthcare spending as a priority. Such attitudes would ultimately increase the pressure on NHS funding [2]. Long waiting times and reduced support for publicly funded health systems are major challenges, and could be exacerbated by the COVID-19 crisis as treatments and elective interventions are postponed [3].

The generally accepted criterion for healthcare waiting time is that patients should be prioritised according to the nature of their clinical condition, rather than by characteristics such as their education, income or nationality [4, 5]. However, there is robust evidence of inequality in this regard, such that patients with a higher socioeconomic status (SES) enjoy privileged access to publicly funded health systems (see [6] and [7], on recent literature in this area).

Sharma et al. [8] and Johar et al. [9] provide strong evidence of inequalities in waiting times for elective surgical procedures, drawing on administrative and census data for Australia. Both studies find that the socioeconomic gradient in waiting times persists even after controlling for possible sample selection by which richer patients may opt for the private sector if they fear a long wait for public sector attention. Similarly, a recent study of the health system in Denmark [5], found significant inequalities in waiting times for certain procedures (e.g., 9–17 days’ longer wait for cataract surgery for less favoured individuals), mostly explained by geographical and institutional factors affecting the hospitals concerned. These authors also revealed important differences for non-Western immigrants, whose average waiting time for health care was 11–26% more than that for Danish nationals.

Regarding specialist consultations and non-emergency surgery within the public sector, an analysis of survey data from European older adults [10], obtained evidence of inequality in favour of the most educated patients in nine European countries, together with a moderate effect of personal income. However, the socioeconomic gradient was considerably larger for specialist consultations than for non-emergency interventions. In this line too, Schoen et al. [11] examined health systems in Australia, New Zealand, Canada, USA and five European countries and found that, on average, waiting times to see a specialist were around two months less for patients who were financially better off. However, there was no SES gradient for elective surgery.

Abásolo et al. [12] investigated specialist waiting times within the Spanish NHS, drawing on data from the 2006 Spanish National Health Survey. These authors showed that an increase of 10% in household income was associated with a fall of 2.6% in waiting times for diagnostic visits. Education also seemed to play an important role in this respect, with more highly educated patients waiting 28% less time.

Overall, most previous research suggests that patients with a lower SES are at far greater risk of experiencing longer waiting times, especially for specialist visits. However, there is only limited evidence of inequalities in waiting times for primary care. Drawing on survey data, Roll et al. [13] analysed the impact of income and type of insurance on waiting time for GP and specialist consultation in Germany. This study provides strong evidence of inequalities in access to both specialist and primary care services. A recent multi-country study by Martin et al.  [14] found evidence of strong disparities in access to primary care in Canada, Norway and Sweden, according to household income.

In the present study, pooled cross-sectional data for GPs and specialist consultations within the Spanish NHS are used to test whether outpatient waiting times depend on SES, measured by education and labour market status. We also determine how much of the SES gradient remains after controlling for nationality and other sociodemographic differences.

This study contributes to the literature in the following ways. First, in contrast to much previous work, we consider not only specialist care, but also primary health care, thus examining whether inequalities start with the patient’s first contact with the health system. Second, the primary dependent variable is continuous, which enables us to implement quantile regression (QR) estimation techniques to examine whether the SES determinants of waiting times vary within the distribution of waiting times. QR techniques are particularly useful for exploring the possible existence of selection bias in specialist care, a factor that might lead to inequalities being overestimated and one that has been relatively neglected in earlier work. While some papers have considered selection bias in inpatient care, we believe ours is the first to empirically address this issue for specialist services. Third, also in contrast to much previous research in this field, our dependent variable is waiting times for medical visits within a universal health system that is specifically mandated to deliver equal treatment for equal need. Moreover, our abundant dataset includes a proxy for the use of private health care, which is useful as an alternative test for possible selection bias. This question is of great significance to health system policymakers, because if the SES gradient can be explained by sample selection, then it should not be interpreted as evidence of systemic inequality. Fourth, we look deeper into inequalities in waiting times on the basis of SES by analysing potentially-relevant factors which have been omitted or underestimated in earlier research, such as nationality status and language fluency [5, 15].

Our results indicate the existence of a SES gradient in waiting times for specialist services, mainly due to differences in education. We show that patients with university studies wait on average 10.6–18.6.7% (9–16 days) less than those with no educational qualifications. However, for GP visits, the hypothesis of a negative SES gradient in waiting times is not strongly supported by our results. Additionally, while the SES gradient flattens with longer waiting times, our findings do not provide evidence that reliance on private health care drives the association between SES and waiting times for specialist care. Finally, our findings appear to be consistent, according to the robustness checks applied.

The remainder of this paper is organised as follows: in “Data and methods” section we present the data source and the main characteristics of the Spanish NHS, after which we set out the empirical strategy employed. The study findings are detailed in “Results” section, and discussed in detail in “Discussion” section. In the final section, we present the main conclusions drawn and identify relevant policy implications.

## Data and methods

### Institutional setting

Spain has a universal health care system that is to a large extent free at the point of delivery [16]. Health services are mostly publicly provided (public healthcare accounted for 70.8% of total health spending in 2019) [1].

While the Spanish NHS provides universal health care to all individuals residing in the country and is funded mainly by general taxation, the proportion of the population covered by private insurance is rapidly increasing, with out-of-pocket spending accounting for 21.8 percent of total expenditure and voluntary health insurance accounting for 7.9 percent [1]. To a large extent, private insurance in Spain provides either a larger choice of providers or a faster access to health care services (duplicate insurance), a feature which is also shared by many OECD countries [1].

Under the Spanish NHS, individuals visit first the primary health care doctor with whom they are registered to make an appointment following the onset of any symptoms [16]. As in most universally provided health systems, GPs act as gatekeepers and decide whether specialised care is necessary. In addition to in-person appointments in health centres, patients have usually access to web or telephone-based services to get appointments for the GP and specialised care.

### Data source

This study is based on microdata drawn from the Spanish Health Barometer (SHB) survey for the years 2010–2019. The SHB survey [17] is conducted annually, with a representative sample of the Spanish population, aged 18 and above, totalling more than 7,800 people per year, and collects information on opinions, attitudes, utilisation and perceptions of health services. Table 1 shows the characteristics of the study sample, which was composed of 24,935 individuals who had paid at least one visit to a GP and of 6,825 who had visited a specialist doctor within the Spanish NHS. The average waiting time for primary care was 3.36 days while for specialist care it was 88.03 days.

**Table 1:** Sample characteristics

Variable	Mean	SD	Min	Max	Missings (%)	Period
Waiting time (days)						
Gp doctor (*n* = 24,935)	3.36	3.79	1	97	3.39%	2010–2019
Specialist doctor (*n* = 6,825)	88.03	92.68	1	1440	5.48%	2011–2013
Education					0.17%	2010–2019
No qualification	6.90%	0.25	0	1		
Primary studies	22.73%	0.42	0	1		
Secondary studies	47.98%	0.50	0	1		
Bachelor studies	22.40%	0.42	0	1		
Laboral status					0.21%	2010–2019
Employed	39.70%	0.4925	0	1		
Managers (private and public institutions)	2.00%	0.14	0	1		
Technical and profesional scientists and intellectuals	6.20%	0.24	0	1		
Support technicians and professionals	6.00%	0.24	0	1		
Office workers	1.80%	0.13	0	1		
Hospitality and shop workers	8.80%	0.28	0	1		
Security and protection workers	0.50%	0.07	0	1		
Qualified workers in the agricultural industry	1.20%	0.11	0	1		
Artisans and qualified workers in the industry, building and mining sectors	4.60%	0.21	0	1		
Industrial machinery and installations' operators	4.00%	0.20	0	1		
Unqualified workers	4.60%	0.21	0	1		
Non-employed						
Retirement pensioner	27.22%	0.45	0	1		
Inactive	12.44%	0.33	0	1		
Unemployed	18.91%	0.39	0	1		
Household income					28.31%	2010–2019
Q1 [0–600 monthly euros]	8.40%	0.28	0	1		
Q2 [601–1,200 monthly euros]	39.20%	0.49	0	1		
Q3 [1,201–2,400 monthly euros]	38.20%	0.49	0	1		
Q4 [2,401- 4,500 monthly euros]	12.20%	0.33	0	1		
Q5 [More than 4,500 monthly euros]	2.00%	0.14	0	1		
Personal income					25.90%	2015–2019
Q1 [0–600 monthly euros]	28.70%	0.452	0	1		
Q2 [601–1,200 monthly euros]	29.20%	0.455	0	1		
Q3 [1,201–2,400 monthly euros]	15.20%	0.359	0	1		
Q4 [2,401- 4,500 monthly euros]	1.30%	0.115	0	1		
Q5 [More than 4,500 monthly euros]	0.20%	0.042	0	1		
Age					0.01%	2010–2019
18 to 35	24.55%	0.43	0	1		
36 to 45	19.23%	0.39	0	1		
46 to 65	32.59%	0.47	0	1		
66 to 75	13.41%	0.34	0	1		
76 or plus	10.22%	0.30	0	1		
Severity						
Chronic diseases	37.23%	0.48	0	1	0.45%	2011–2019
Self-reported health status					0.22%	2010–2019
Excellent	12.65%	0.33	0	1		
Good	54.17%	0.50	0	1		
Average	27.44%	0.45	0	1		
Poor	4.85%	0.21	0	1		
Worst	0.89%	0.09	0	1		
Living area					0.00%	2010–2019
Rural (Hab < 10,000)	20.82%	0.41	0	1		
Gender					0.00%	2010–2019
Female	56.01%	0.50	0	1		
Immigrant status					0.05%	2010–2019
Spanish only	90.80%	0.24	0	1		
Double Spanish nationality	3.21%	0.18	0	1		
European Union	1.74%	0.13	0	1		
Rest of Europe	0.30%	0.05	0	1		
Latin American	2.90%	0.17	0	1		
North American	0.04%	0.02	0	1		
African	1.04%	0.10	0	1		
Asian	0.10%	0.03	0	1		
Fluency					0.90%	2010–2019
Low fluency	0.13%	0.04	0	1		
Medium fluency	3.01%	0.17	0	1		
High fluency	96.87%	0.17	0	1		
Utilization of Spanish National Health System						2010–2019
Gp doctor (number of visits)					0.04%	
1	20.00%	0.40	0	1		
2	23.80%	0.43	0	1		
3 or more	50.80%	0.50	0	1		
Specialist doctor (number of visits)					0.08%	
1	44.50%	0.50	0	1		
2	27.60%	0.45	0	1		
3 or more	28.00%	0.45	0	1		
Utilization of private healthcare						
Gp doctor (number of visits)					0.04%	
0	93.50%	0.25	0	1		
[1, 2]	4.60%	0.21	0	1		
[3 or more)	1.90%	0.14	0	1		
Specialist doctor (number of visits)					0.08%	
0	89.80%	0.30	0	1		
[1, 2]	7.20%	0.26	0	1		
[3 or more)	3.10%	0.17	0	1		

### Study variables

#### Waiting time

Waiting time to see a GP in the Spanish NHS is measured as the number of days elapsed since the appointment was made until the medical visit took place, during the twelve months prior to the survey. For specialist consultations, waiting time is defined as the time elapsed since the GP’s referral until the patient was seen by the specialist. The main dependent variables are based on the questions:

*“The last time you requested a consultation with your doctor or general practitioner, how long did it take from the day you requested the appointment to the day the consultation took place?”*

*“The last time your doctor or general practitioner referred you to the specialist, how long did it take from the day you made an appointment with the specialist until the specialist saw you?”*

Only visits within the Spanish NHS are taken into account for our analysis. We exclude patients who made no appointment due to the emergency nature of the visit and were attended on the same day. ﻿The time frame considered is restricted to those years with homogeneous information on waiting time in the survey (i.e., 2010–2019). Additionally, for specialist care, our focus is restricted to the 2010–2013 period since information on speciality type is only available for that period.

#### Independent variables

Our core socioeconomic variables are the patients’ education and employment status. Education is split into four categorical variables (no qualifications; primary studies; secondary studies; university studies). Activity status is also categorised into four variables (employed; inactive; retired; unemployed), with employment status subdivided into nine additional groups on the basis of the National Classification of Occupations (CNO-2011) In addition, we have included household income categorised into five quintiles ranging from 0–600 monthly euros to over 4500 euros (see Table 1).^1^ However, for the sake of simplicity, given the high number of employment variables, and to make the most of our estimating sample (as household income has a high number of missing values, see Table 1), we have used education and employment status (aggregated into four categories) as the baseline SES indicators and have included the estimations with the additional SES variables in Tables 2–6 of the Supplementary Material 2.

Severity of the patient’s condition is measured by age, self-assessed health status and the presence of chronic diseases. Since severe health conditions might be correlated (negatively) with waiting time and SES, failure to control for this parameter might generate biased results. Age is assigned to one of five categories (ranging from 18–35 years to 76 years or older). Self-assessed health status ﻿is measured by a categorical indicator by which general health is considered to be “excellent” “good”, “fair”, “bad” or “very bad’. Finally, a dummy variable controls for the presence of any chronic conditions. The number of visits to a publicly-funded specialist doctor is categorised into three groups (one, two and three or more consultations) to proxy either the type of consultation diagnosis (first visit) or a review (subsequent visits) for specialist care or to address the clinical need.

Our analysis also controls for the patient’s gender, size of area of residence, citizenship status and fluency in Spanish. Nationality has received little attention in the literature, with the exception of Simonsen et al. [5] and Tinghög et al. [15]. Differences in citizenship status may be a source of inequality in waiting times. Thus, studies such as Simonsen et al. [5] and Jiménez-Rubio and Hernández-Quevedo [18] have documented inequalities not only in access but also in waiting times according to the patient’s nationality. Finally, we also include the number of visits to the private GP or specialist doctor, to take into account the possibility that patients might seek private healthcare if NHS attention is subject to long waiting times. This option might bias our inequality estimates by generating an apparent negative gradient between SES and waiting time for patients receiving treatment within the NHS.

### Analysis technique: specification and estimation

#### Empirical specification

We model the waiting time for primary or specialist care visits ${w}_{ijt}$ for patient *i* within the NHS in region *j* in year *t* as:

1 $$\mathrm{ln}\left({W}_{ijt}\right)={\beta }_{0}+{\beta }_{1}SE{S}_{ijt}+{\beta }_{2}Se{v}_{ijt}+{\beta }_{3}{Z}_{ijt}+{d}_{j}+{d}_{t}+{e}_{ijt}$$

where $$SE{S}_{ijt}$$ is a set of variables measuring SES; $$Se{v}_{ijt}$$ is a vector of variables measuring severity; $${Z}_{ijt}$$ denotes the effect of other explanatory variables which may explain the SES gradient; $${d}_{j} \ \mathrm{and}\ \ {d}_{t}$$ represent region and year effects, respectively; and $${e}_{ijt}$$ is the error term. Since waiting times are highly skewed to the left, the dependent variable is transformed into the log form. For specialist health care, we also control for specialty fixed effects ($${d}_{s})$$.

Our main coefficient of interest, ${\widehat{\beta} }_{1}$, measures the association between patients’ SES and the waiting time for publicly funded primary and specialist visits. We also control for severity in order to eliminate possible bias in this respect, since health status and SES may be correlated. Our initial assumption is that more severe patients will have shorter waiting times [5, 10]. Severity is measured by the patient’s age and by the presence of chronic illness.

Our extended models demonstrate how much of the SES gradient remains after controlling for (i) additional individual attributes (gender and area of residence), (ii) citizenship status and fluency with the language and (iii) utilisation of private health services, all of which may be relevant determinants of access to health services. Appendix 1 details the role played by citizenship status and fluency in Spanish in explaining differences in waiting times; we assume that greater fluency will help the patient to better navigate the health system.

In this analysis, we employ ordinary least squares (OLS) and robust standard errors clustered at region level. In addition, we include sampling weights throughout to make the sample as representative as possible of the Spanish population. We are cautious about interpreting our results as causal relationships, since the assumption of exogeneity does not hold for SES and waiting time, because we cannot control unobserved factors such as health illiteracy, which may be correlated with both waiting time and SES [5]. However, the inclusion of variables such as language fluency and immigrant status together with the longitudinal nature of our data are expected to improve the reliability of our analysis.

#### Quantile regression

Since the relationship between SES and waiting times may vary at different points of the waiting time distribution, we make use of quantile regression (QR), in line with previous research (see e.g. Sharma et al., [8]). Accordingly, Eqs. (1) and (2) can be rewritten for the τth conditional quantile (Qτ) as follows [19]:

2 $${Q}_{\tau }(\mathrm{ln}\left({W}_{ijt}\right))={\beta }_{0}(\tau )+{\beta }_{1}(\tau )SE{S}_{ijt}+{\beta }_{1}(\tau )Se{v}_{ijt}+{\beta }_{3}{(\tau )Z}_{ijt}+{d}_{j}(\tau )+{d}_{t}(\tau )+{e}_{ijt }(\tau )$$

where ${\beta }_{\tau }$ represents the slope coefficients and ${e}_{\tau }$ is the idiosyncratic error term at the τth conditional quantile. Quantile regressions are based on minimising the sum of the weighted absolute values of waiting times. This approach has the following advantages: (1) it allows the effect of the explanatory variables to vary within the distribution of waiting times; (2) it is robust to outliers in the observations of waiting times. The command *sqreg* was performed to estimate values of q in Stata 15 [20] and to test various hypotheses about our dependent variables.

## Results

### Inequalities in waiting times for primary care

Table 2 presents the OLS estimations obtained for waiting times in primary care. Model 1 includes SES (measured by education and activity status), gender, severity (proxied by the presence of chronic disease) and age. Model 2 includes the following additional control variables; type of area of residence (rural vs. urban); citizenship status; fluency in Spanish and utilisation of primary services in private healthcare.

**Table 2:** OLS estimations for waiting time in primary care. 2011–2019

Waiting times in primary care	Model 1	Model 2
Education		
No qualifications	Ref	
Primary studies	0.003	-0.007
	(0.029)	(0.023)
Secondary studies	0.011	-0.009
	(0.030)	(0.023)
University studies	0.000	-0.031
	(0.031)	(0.022)
Employment status		
Inactive	Ref	
Retirement pensioner	-0.005	-0.003
	(0.013)	(0.014)
Unemployed	-0.012	-0.010
	(0.014)	(0.017)
Employed	0.004	0.004
	(0.025)	(0.026)
Gender		
Male	Ref	
Female	0.043***	0.038***
	(0.011)	(0.011)
Severity		
Age (years)		
18 to 24	Ref	
35 to 44	-0.050***	-0.049***
	(0.010)	(0.010)
45 to 64	-0.042***	-0.041***
	(0.009)	(0.010)
65 to 75	-0.064***	-0.066***
	(0.015)	(0.017)
75 or more	-0.129***	-0.132***
	(0.026)	(0.026)
Chronic illness		
Presence of chronic illness	0.058***	0.053***
	(0.012)	(0.013)
GP visits		
1 visit	Ref	
2 visits	0.029*	0.030*
	(0.016)	(0.017)
3 or more visits	0.038*	0.040*
	(0.018)	(0.019)
Area of residence		
Urban	Ref	
Rural		-0.236***
		(0.043)
Citizenship status		
Native Spanish	Ref	
Spanish acquired		0.060***
		(0.016)
Foreign		0.033
		(0.028)
Fluency		
High	Ref	
Medium		-0.066**
		(0.029)
Low		-0.105
		(0.072)
Private healthcare visits		
0 visits	Ref	
1 to 2 visits		0.006
		(0.020)
3 or more visits		0.128**
		(0.049)
Region fixed effects	Yes	Yes
Year fixed effects	Yes	Yes
Constant	0.893***	0.945***
	(0.052)	(0.046)
Observations	24,935	24,935
R-squared	0.249	0.261
AIC	51325	50919
BIC	51455	51049

^1^Our individual of reference is a male with no studies; inactive; between 18 and 34 years old; no chronic disease; no visit to private GPs; Spanish; fluent and living in an urban area

^2^Model 1: SES, severity variables and time and region fixed effects. M2: M1 + area of residence + immigrant status + Spanish fluency

^3^Double nationality: Spanish nationality and other

^4^Standard errors are shown in parentheses. *** *p* < 0.01, ** *p* < 0.05, * *p* < 0.1

Our analysis revealed significant differences in waiting times for primary care according to gender, severity and area of residence. On average, women waited around 4% longer than men. Regarding SES, while the baseline model revealed no evidence of important differences in this respect, the estimations in Tables 1 and 2 of the Supplementary Material provided suggestive evidence of a SES gradient in favour of moderate to highly qualified workers (approximately 8% less wait). Moreover, waiting times for primary care were slightly longer for chronically ill patients and for private insurance holders. In particular, patients who reported at least one chronic disease, and those who were frequent users of private healthcare services were more likely to experience longer waiting times. On the other hand, waiting times in primary care were shorter for older patients. Interestingly, those who lived in rural areas waited considerably less than their counterparts in urban areas (approximately 25% less wait or 0.8 days).Moreover, we found statistically significant differences in waiting time for double nationality individuals albeit moderate in size (about 6% more wait, or 0.2 days) (see Table 2 and Supplementary Material 1). Finally, according to Fig. 1 in the Supplementary material only four regions Cataluña, Canarias, Comunidad Valenciana and Islas Balearesare above the reference region in terms of primary care wait.

**Fig. 1:**
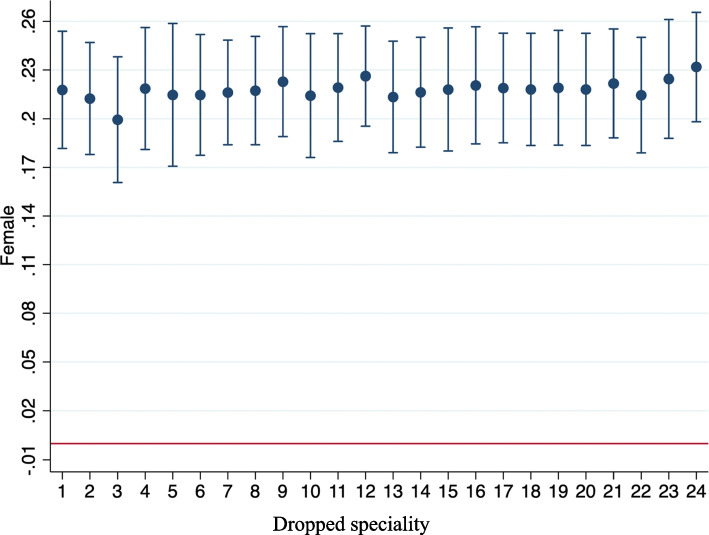
Leaving-out test for female gender, across specialities 1. General and digestive surgery 2. Obstetrics and gynaecology 3. Ophthalmology 4. Otorhinolaryngology 5. Traumatology and orthopaedic surgery 6. Urology 7. Neurology 8. Digestive system 9. Circulatory system 10. Dermatology (skin diseases) 11. Pneumology (respiratory system) 12. Medical oncology 12. Psychiatry 13. Rehabilitation 14. Endocrinology 15. Allergology 16. Cardiovascular surgery 17. Geriatrics 18. Haematology 19. Haemotherapy 20. Immunology 21. Internal medicine 22. Nephrology 23. Rheumatology 24. Other

The Akaike Information Criterion (AIC) and the Bayesian Information Criterion (BIC) were calculated to compare the goodness-of-the-fit^2^ of each model considered. According to these test results and the ICC values obtained, model 2, which represents the extended model, performs best.

### Inequalities in waiting times for specialist visits

Table 3 shows the OLS estimations obtained for waiting times for specialist attention. The results show the presence of a robust SES gradient, mostly on the basis of education and, to a lesser extent, employment status. According to Model 2, the most highly educated patients wait 18.6% (16.4 days for a sample mean of 88.03 days) less than those with no qualifications. Regarding activity status, unemployed patients wait 12.9–13.7% (12 days) more than employed patients. With respect to the alternative SES indicators employed, substantial differences were found in waiting times on the basis of both employment type and household income, reinforcing our baseline results (see Tables 2 and 4 of the Supplementary Material). It is important to remark, however, that the size of the education effect attenuates considerably when household income is included in the estimations, and that the magnitude of the effect is considerably high for highly skilled workers (around 19% less wait). In addition, our results provide suggestive evidence of differences on the basis of health, with an average additional wait of 19.4–19.2% (17 days) for individuals diagnosed with a chronic condition.

**Table 3:** OLS estimations for waiting times for specialist care. 2011–2013

Waiting times in specialist care	Model 1	Model 2
Education		
No qualifications	Ref	
Primary studies	-0.081	-0.085
	(0.055)	(0.055)
Secondary studies	-0.158***	-0.163***
	(0.037)	(0.038)
University studies	-0.169***	-0.186***
	(0.041)	(0.043)
Employment status		
Inactive	Ref	
Retirement pensioner	0.090	0.092
	(0.089)	(0.088)
Unemployed	0.129***	0.137***
	(0.042)	(0.043)
Employed	0.044	0.046
	(0.054)	(0.054)
Gender		
Male	Ref	
Female	0.195***	0.196***
	(0.028)	(0.029)
Severity		
Age (years)		
18 to 24	Ref	
35 to 44	0.023	0.023
	(0.038)	(0.039)
45 to 64	0.012	0.002
	(0.030)	(0.028)
65 to 75	0.005	-0.010
	(0.053)	(0.052)
75 or more	-0.191***	-0.204***
	(0.059)	(0.057)
Chronic illness		
Presence of chronic illness	0.194***	0.192***
	(0.037)	(0.037)
Specialist visits		
1 visit	Ref	
2 visits	0.030	0.032
	(0.030)	(0.029)
3 or more visits	-0.176***	-0.172***
	(0.041)	(0.040)
Area of residence		
Urban	Ref	
Rural		-0.027
		(0.042)
Citizenship status		
Native Spanish	Ref	
Spanish acquired		-0.158
		(0.133)
Foreign		-0.103
		(0.077)
Fluency		
High	Ref	
Medium		0.005
		(0.136)
Low		-0.334
		(0.570)
Private healthcare visits		
0 visits	Ref	
1 to 2 visits		0.167**
		(0.077)
3 or more visits		0.018
		(0.071)
Region fixed effects	Yes	Yes
Year fixed effects	Yes	Yes
Specialist FE	Yes	Yes
Constant	3.641***	3.650***
	(0.069)	(0.074)
Observations	6,825	6,825
R-squared	0.045	0.047
AIC	21556	21541
BIC	21665	21650

^1^Our individual of reference is a male with no studies; inactive; between 18 and 34 years old; no chronic disease; one visit to public GPs; no visit to private GPs; Spanish; fluent and living in an urban area

^2^Model 1: SES severity variables and region, year and specialties’ fixed effects. M2: M1 + area of residence + immigrant status + Spanish fluency. Model 3 also control for other non-observable effects at time or region level

^3^Double nationality: Spanish nationality and other

^4^Standard errors are shown in parentheses. *** *p* < 0.01, ** *p* < 0.05, * *p* < 0.1

Among the other control variables, a gender gap was also found for specialist healthcare, with female patients having to wait 19.4–19.5% (17 days) longer than men, even after controlling for SES and severity. On the other hand, patients who paid 1 or 2 visits to private healthcare suppliers faced a slightly longer wait. Interestingly, marked differences were also found according to nationality, with EU nationals reporting substantially shorter waiting times (around 22%, see Table 1 in Supplementary Material). Finally, some interesting differences were found regarding the region of residence, with Madrid and La Rioja being the regions with less wait in comparison to the region of reference (Andalucía) (see Fig. 2 in the Supplementary Material).

According to the goodness-of-the-fit measures of each model considered, Model 2, which controls for area of residence, nationality and utilisation of private healthcare, performs best.

### Inequalities in waiting times across the distribution

Table 4 shows the quantile regressions obtained for waiting times for specialist care. Columns 2–6 show the estimates for the 10^th^, 25^th^, 50^th^, 75^th^ and 90^th^ quantiles. These results illustrate differences in the distribution of waiting times for specialist healthcare services, with respect to education background, especially in moderate levels of the waiting distribution (quantile 25^th^). There is also evidence of socioeconomic differences according to employment status, also mostly in moderate segments as well (the 25^th^ quantile). Moreover, there were important differences in the distribution of waiting times for specialist consultations when the patients presented chronic diseases (longer waiting times) and for higher numbers of visits (shorter waiting times). Finally, our baseline findings, and our estimations based on alternative SES covariates (Tables 3 and 5 of the Supplementary Material) suggest that there is evidence of greater use of private consultations when waiting times for Spanish NHS services are longer (the 50^th^ and 75^th^ quantiles).

**Table 4:** Quantile regression for waiting times for specialist care (2011–2013)

Waiting time for specialist	Q(0.10)	Q(0.25)	Q(0.50)	Q(0.75)	Q(0.90)
Education					
Primary	-0.132	-0.137	0.0076	-0.0272	0.0484
Secondary	-0.273	-0.292**	-0.0872	-0.0793	0.00000643
University	-0.198	-0.279*	-0.163*	-0.138	-0.0832
Employment status					
Retired	0.0059	0.0901	0.095	0.119	0.0693
Unemployed	0.219	0.173**	0.0876	0.119*	0.0883
Employed	-0.0839	0.0613	0.0336	0.101*	0.107
Gender					
Female	0.272***	0.203***	0.168***	0.163***	0.0919***
Age (years)					
35 to 44	0.0672	0.00655	0.00591	-0.0482	-0.111
45 to 64	0.147	-0.0243	-0.0154	-0.05	-0.0659
65 to 74	0.00495	-0.007	-0.077	-0.0326	-0.037
75 or more	-0.121	-0.328*	-0.186*	-0.166	-0.0793
Chronic illness					
Presence of chronic illness	0.111	0.177***	0.210***	0.196***	0.166***
Number of visits to GP					
2 visits	0.260***	0.135*	0.0496	-0.0202	-0.0831*
3 or more visits	0.00921	-0.0911	-0.148***	-0.249***	-0.256***
Area of residence					
Rural	-0.0599	-0.0501	-0.0517	0.0199	0.0186
Citizenship status					
Spanish	-0.0694	-0.231	-0.00478	-0.0968	-0.161
Foreign	-0.285	-0.0824	-0.145	-0.183**	-0.201*
Private consultations					
1 or 2 visits	0.121*	0.161*	0.146**	0.154*	0.053
3 or more visits	-0.45	0.00697	0.145	0.0977	0.0126
Fluency					
Medium	0.0619	-0.063	0.159	0.137	0.235
Low	-1.662	-0.915	-0.0364	0.132	-0.0103
Specialist fixed effects	Yes	Yes	Yes	Yes	Yes
Year fixed effects	Yes	Yes	Yes	Yes	Yes
Region fixed effects	Yes	Yes	Yes	Yes	Yes
Constant	3.636***	3.221***	3.788***	4.313***	4.968***

^1^Our individual of reference is a male who has visited Traumatology services; with no studies; inactive; between 18 and 34 years old; with no chronic disease; one visit to public specialist care; no visit to a private specialist doctor; Spanish citizenship; fluent and living in an urban area

^2^Full model specification: SES and severity variables + area of residence + immigrant status + fluency + private healthcare utilization + region and -year fixed effects

^3^*** *p* < 0.01, ** *p* < 0.05, * *p* < 0.1

### Robustness tests

Several robustness checks were performed. First, region-specific time trends were included to take into account potential differential trends in the outcome variables across regions. Table 5 shows that this flattens the SES gradient for specialist care, but the results vary only marginally. Second, the models were implemented with the addition of self-assessed health (SAH) as another control variable. As can be seen in Table 6, adding SAH affected neither the statistical significance nor the size of the rest of variables for waiting times for primary or specialist care. However, only marginally significant effects of SAH were found for waiting times in primary care. Therefore, it was not included in the baseline model.

**Table 5:** OLS estimations for primary and specialist services, controlling for region-specific time effects

Waiting times	Primary care	Specialist care
Education		
No qualifications	Ref	
Primary studies	-0.005	-0.085
	(0.022)	(0.055)
Secondary studies	-0.006	-0.157***
	(0.023)	(0.040)
University studies	-0.028	-0.179***
	(0.021)	(0.046)
Employment status		
Inactive	Ref	
Retirement pensioner	-0.002	0.094
	(0.015)	(0.088)
Unemployed	-0.011	0.134***
	(0.017)	(0.045)
Employed	0.002	0.042
	(0.026)	(0.054)
Region fixed effects	Yes	Yes
Year fixed effects	Yes	Yes
Region-Trend	Yes	Yes
Specialist fixed effects	No	Yes
Constant	0.899***	3.640***
	(0.037)	(0.068)
Observations	24,935	6,825
R-squared	0.263	0.054
AIC	50829	21493
BIC	50959	21602

^1^For primary care, our individual of reference is a male with no studies; inactive; between 18 and 34 years old; no chronic disease; one visit to public GPs; no visit to private GPs; Spanish citizenship; fluent and living in a urban area. For specialist consultations, our individual of reference is a male who has visited Traumatology services; with no studies; inactive; between 18 and 34 years old; with no chronic disease; one visit to public specialist services; no visit to private specialist doctor; Spanish and living in a urban area

^2^Full model specification: Only SES variables are reported

^3^*** *p* < 0.01, ** *p* < 0.05, * *p* < 0.1

**Table 6:** OLS estimations for waiting time for primary and specialist care, controlling for self-assessed health

Waiting times	Primary care	Specialist care
Education		
No qualifications	Ref	
Primary studies	0.004	-0.086
	(0.023)	(0.060)
Secondary studies	0.007	-0.155***
	(0.023)	(0.051)
University studies	-0.011	-0.169**
	(0.023)	(0.060)
Employment status		
Inactive	Ref	
Retirement pensioner	-0.006	0.083
	(0.014)	(0.091)
Unemployed	-0.010	0.128**
	(0.017)	(0.045)
Employed	0.006	0.039
	(0.026)	(0.054)
Severity		
Self-assessed health status		
Very bad	Ref	
Poor	-0.101	0.194
	(0.080)	(0.131)
Average	-0.139*	0.204
	(0.077)	(0.151)
Good	-0.194**	0.149
	(0.076)	(0.155)
Excellent	-0.203**	0.012
	(0.071)	(0.190)
Chronic illness	0.029**	0.166***
	(0.012)	(0.050)
Region fixed effects	Yes	Yes
Year fixed effects	Yes	Yes
Region-Trend	Yes	Yes
Specialist fixed effects	No	Yes
Constant	1.124***	3.518***
	(0.087)	(0.156)
Observations	24,900	6,814
R-squared	0.262	0.049
AIC	50746	21495
BIC	50875	21604

^1^For primary care, our individual of reference is a male with no studies; inactive; between 18 and 34 years old; no chronic disease but very bad self-reported health status; one visit to public GOs; no visit to private GPs; Spanish and living in an urban area. For specialist consultations, our individual of reference is a male who has visited Traumatology services; with no studies; inactive; between 18 and 34 years old; with no chronic disease but very bad self-reported health status; one visit to public specialist doctor; no visits to private specialist doctor; Spanish and living in an urban area

^2^Full model specification: SES and severity variables + area of residence + immigrant status + private healthcare utilization + region-year fixed effects

^3^*** *p* < 0.01, ** *p* < 0.05, * *p* < 0.1

Third, by means of a leaving-out test for the different specialties, we tested the hypothesis that the gender gap for waiting times may be driven by related differences in the use of specialist healthcare (for example, gynaecological attention). However, as Fig. 1 shows, the gender gap remained throughout the sample. However, when observations for ophthalmology were omitted, the gender differences decreased slightly.

## Discussion

The main aim of the study described in this paper is to determine empirically whether there exists a SES gradient for waiting times in primary and specialist care within the Spanish NHS for patients presenting the same level of severity. Using rich data from the Spanish Health Barometer and making use of OLS and quantile regression, we examine how much of the SES gradient remains after controlling for potentially relevant confounding variables such as level of severity, utilisation of private healthcare services and nationality.

Although most previous studies in this field have focused on waiting times in inpatient care, the presence of a SES gradient in access to health services may originate in primary care and continue throughout the patient's experience with the health system. The channels which may account for this gradient include differences in the availability of GPs according to the patient’s place of residence, in access to networks or in employment flexibility (usually more available to the more highly educated) [14]. Although we did not detect strong inequalities arising from the patients’ household income in primary care, we did find some modest effects on the basis of employment status, which are in line with recent research conducted in several OECD countries [14]. Another study, by Roll et al. [13], drawing on German data, found that for patients who earn 2,000 or more euros monthly, the waiting time for primary care was one day shorter than for those on lower incomes. Our own analysis provides evidence of inequalities in waiting time to see a GP on the basis of gender (favouring males), severity (pro-healthy gradient) and area of residence (favouring rural areas). The latter result may be explained by the fact that rural areas have relatively more healthcare facilities (especially outpatient clinics) and because access to these is easier than to similar facilities in urban areas [12]. Previous studies have detected a gender gradient in waiting time, attributing this to different patterns of service utilisation between men and women. Furthermore, differences in severity, and the fact of shorter waiting times for men, may be due to the latter presenting a more advanced stage of the disease when they contact the health care provider.

Regarding specialist consultations, evidence of SES-related inequalities in waiting times persists even after adjusting for severity and other potential confounding factors. This finding corroborates the earlier work of Siciliani and Verzulli [10] and Abásolo et al. [12] in this respect. While Siciliani and Verzulli [10] found modest effects for income but substantial ones for education (68% less waiting time according to education background), Abásolo et al. [12] showed that patients with only primary studies had to wait 28% longer than those with university studies. In line with this previous work, we show that patients with university studies wait around 19% (approx. 16 days) less than those with no educational qualifications.

Concerning other relevant explanatory variables, we found that patients who had a chronic illness were likely to wait longer for primary or specialist attention, despite their higher level of severity. In this respect, Abásolo et al. [12], Roll et al. [13] and Carrière and Sanmartin [21] argue that the treatments for chronic illness may be more complex, thus provoking increased waiting time. With respect to nationality, we found evidence of a considerate pro-immigrant gradient for waiting times in specialist consultations, favouring EU nationals (whose average waiting time was 24% less). The opposite result was found for elective surgery in Denmark [5] but not in Sweden, where active workforce immigrants waited around 41% less for gynaecology services [15]. Interestingly, we detected a slightly longer waiting time (5.7%) for primary care for those citizens who hold the Spanish nationality and other.

Various factors might account for the inequalities found favouring relatively more educated patients. Firstly, they have lower transaction costs (with better-informed networks, better information about how the NHS works and lower travelling costs) [10] and are more engaged with the health system, perhaps because those who are better educated make a more convincing case for higher priority or because they better articulate their needs and thus are more persuasive to the GP [22]. In addition, better-educated patients may work in more flexible jobs and thus have greater freedom to attend the first available medical appointment. Finally, the doctors consulted might be subject to prejudices regarding health behaviour, thus giving rise to inequalities in the attention provided.

Quantile regressions were performed to determine whether the effects of the study variables varied across the distribution of waiting times for specialist care. This analysis revealed marked SES inequalities across the distribution of waiting times, which is consistent with our finding that more frequent users of private health care experience longer waiting times for NHS attention than those making less use of the private system. Interestingly, this frequency of use seems to better capture the potential self-selection issue than a dummy for private insurance, an approach that has been employed in earlier studies (e.g. Abásolo et al., [12]). However, in line with Sharma et al. [8] and Johar et al. [9], we find that regardless of self-selection, there is a strong SES gradient for specialist services, concentrated among moderate levels of the waiting time distribution.

## Conclusions

There is evidence that waiting times for limited healthcare resources are not assigned equitably among patients. This issue is of particular importance in the current global crises – COVID-19 and economic – which could exacerbate these disparities as rising numbers of treatments are being postponed. In contrast to previous studies in this field, we use a rich longitudinal dataset to study the SES gradient in waiting times for both primary and secondary care. Additionally, we examine how much of the SES gradient remains after controlling for nationality and other relevant sociodemographic differences (such as gender and the use of private healthcare), which have been somewhat neglected in the literature. We also contribute new knowledge by investigating whether these differences vary significantly at different levels of waiting times, using quantile estimation techniques.

Our analysis did not detect a strong SES gradient related to education, income or labour market status regarding waiting times for GP visits. However, we did find some evidence of a SES gradient in favour of moderate to high skilled workers. Nevertheless, the results obtained suggest a considerable SES gradient for specialist services, mainly explained by differences in education, employment level and income. In addition, for GP services we find evidence of inequalities according to gender, severity (pro-healthy gradient) and area of residence. A similar gender gap was also found for specialist medical attention. Our quantile estimations show that for women, the SE gradient is stronger the shorter the waiting time is of their condition, the longer they must wait. The fact that men have shorter waiting times in this situation may be due to their presenting more advanced stages of disease and a greater number of pathologies [21]. Interestingly, in contrast to a previous study on elective surgery, in which Western Europeans reported longer waiting times than other nationalities [5], we detected disparities in waiting times for specialist attention according to citizenship status, but favouring EU citizens in their wait for specialist services.

The strengths of this study enable us to draw significant conclusions. For example, we exploit the richness of our data to investigate a potential selection bias in access to healthcare, by which some users of private healthcare systems resort to the NHS only when waiting times are relatively short. This pattern of use would bias the SES gradient. Furthermore, we employ quantile regression to investigate differences within the distribution of results for SES, and also control for the number of private medical consultations to determine whether there is any association between this parameter and waiting times. Our findings strongly suggest that patients make significant use of private healthcare when waiting times for NHS attention are above the median. Nevertheless, there exists a substantial SES gradient for specialist care (favouring the better educated individuals) regardless of the presence or absence of selection bias. Selective waiting time barriers pose an important challenge, especially within universal health care systems, and they may impact negatively on population health, possibly exacerbating existing health inequalities.

This study is subject to certain limitations. First, data for waiting times in private healthcare were not available and so we were unable to adjust for sample selection bias using a Heckman selection type model. However, unlike much previous research, we had access to rich information on waiting times in the NHS and on visits to both publicly and privately funded health services, data which are very useful for investigating potential self-selection and for detecting differences in waiting time distribution according to SES. The second major limitation is that although the dataset provided detailed information about waiting times and other relevant socioeconomic factors, our analysis incorporated only a limited number of need-related variables. This issue might be more significant regarding the situation of waiting time inequalities in primary care, since for specialist visits first and subsequent visits are determined by the health professionals concerned. Lastly, our key variable of waiting times is self-reported. Offsetting potential problems in this regard, our use of rich survey data in this area could have some advantages over the use of administrative records on waiting times, since it avoids the bias that may arise from health providers misreporting waiting times due to political motivations. Nevertheless, it should be noted that survey data is not exempt from problems, such as the lack of continuity in the availability of relevant variables (for instance, in our case in the SHB speciality type is not included after 2013 and personal income is only included after 2014).

Uncovering the sources of inequality in waiting times within the Spanish NHS is crucial to effective policy design, since the reality of rationing by waiting times seems to be less equitable than is desirable. Since the current system by which GPs determine the scheduling of medical attention does not ensure equity in waiting times, we suggest that better-detailed guidelines should be given for referral and that greater transparency in this respect should be provided. For example, there should be more robust and simpler mechanisms for booking (taking into account that on-line scheduling might exacerbate inequalities) and more transparency from hospitals in the form of reports on waiting times for procedures according to SES indicators (such as the patient’s postcode), in order to highlight differences in this regard.

Finally, we believe that further research in this area is needed, for a more comprehensive investigation of the mechanisms underlying potential barriers of access to healthcare, in terms of inequality in waiting times, thus helping policymakers and managers design and implement evidence-informed policies to address existing disparities in access to healthcare.

## Supplementary Information

**Additional file 1: Supplementary file 1.**

## Data Availability

All data is available in open access, https://www.mscbs.gob.es/estadEstudios/estadisticas/BarometroSanitario/home_BS.htm. The codes used for statistical analysis can be requested from the authors.

## Footnotes

1 As an extra SES indicator, we included personal income which was only available in the SHB for the period 2015–2019, and thus was only included in the estimations for primary care (see descriptives for this variable in Table 1 and estimation results when including this variable as a covariate in Table 6 in the Supplementary Material).

2 AIC and BIC are written in the form [-2log*L* + *kp*], where *L* is the likelihood function, *p* is the number of parameters in the model and *k* is 2 for AIC and log(*n*) for BIC. In the case considered, a smaller AIC or BIC indicates a better-fitting model.
