# Supplementary material for: Waiting times in healthcare: equal treatment for equal need?
Source: Int J Equity Health. 2022 Dec 20;21:184. doi: 10.1186/s12939-022-01799-x (PMC9763792; doi:10.1186/s12939-022-01799-x)
Supplement: Supplementary file 1 — Additional file 1: Supplementary file 1. [file 12939_2022_1799_MOESM1_ESM.docx]

**Supplementary material**

**Supplementary material 1** Estimations of waiting times for specialist services with the Spanish NHS by country of origin.

**Table S1** Waiting time estimations for country of origin.

| **Waiting times** | **Primary care** | **Specialist care** |
| --- | --- | --- |
| Native | Ref. | |
| Double nationality | 0.057*** | -0.189 |
|  | (0.017) | (0.154) |
| European Union | 0.009 | -0.224** |
|  | (0.035) | (0.105) |
| Europe | 0.046 | -0.065 |
|  | (0.060) | (0.332) |
| Latin American | 0.028 | -0.109 |
|  | (0.034) | (0.132) |
| North American | -0.106 | -0.314 |
|  | (0.295) | (0.268) |
| African | 0.029 | 0.095 |
|  | (0.057) | (0.205) |
| Asian | 0.081 | -0.209 |
|  | (0.083) | (0.290) |
| High fluency | Ref. | |
|  |  |  |
| Medium fluency | -0.123 | 0.043 |
|  | (0.085) | (0.179) |
| Low fluency | -0.062 | -0.461 |
|  | (0.039) | (0.474) |
| **Specialist FEs** | No | Yes |
| **Regions FEs** | Yes | Yes |
| **Year FEs** | Yes | Yes |
| **Observations** | 24,872 | 6,799 |
| **R-squared** | 0.261 | 0.051 |
| **AIC** | 50789 | 21440 |
| **BIC** | 50919 | 21550 |

^1^ For primary care, our individual of reference is a male with no studies; inactive; between 18 and 34 years old; no chronic disease; one visit to public GP; no visit to private GPs; Spanish; fluent and living in a urban area. For specialist consultations, our individual of reference is a male who has visited Traumatology services; with no studies; employed; between 18 and 34 years old; with no chronic disease; one visit to public GP; no visits to private specialist doctor; Spanish; fluent and living in a urban area.

^2^ Full model specification: SES and severity variables + area of residence+ immigrant status + private healthcare utilization + region-year fixed effects.

^3^ Note: *** p<0.01, ** p<0.05, * p<0.1

**Supplementary material 2** Estimations of waiting times for primary and specialist services with the Spanish NHS by disaggregated employment status.

**Table S2** OLS estimations for waiting including disaggregated employment status

| **Waiting times in specialist care** | **Primary care** | **Specialist care** |
| --- | --- | --- |
|  |  |  |
| **Education** |  |  |
| No qualifications | Ref. | |
| Primary studies | -0.007 | -0.085 |
|  | (0.023) | (0.056) |
| Secondary studies | -0.009 | -0.173*** |
|  | (0.023) | (0.036) |
| University studies | -0.021 | -0.155*** |
|  | (0.023) | (0.052) |
| **Employment status** |  |  |
| Inactive | Ref. | |
| Retirement pensioner | -0.013 | 0.129 |
|  | (0.009) | (0.088) |
| Unemployed | -0.022*** | 0.179*** |
|  | (0.006) | (0.048) |
| Managers (private and public institutions) | -0.026 | 0.060 |
|  | (0.023) | (0.149) |
| Technical and profesional scientists and intellectuals | -0.083*** | -0.003 |
|  | (0.021) | (0.102) |
| Support technicians and professionals | 0.002 | -0.007 |
|  | (0.036) | (0.084) |
| Office workers | 0.004 | 0.217 |
|  | (0.045) | (0.155) |
| Hospitality and shop workers | -0.001 | 0.092 |
|  | (0.022) | (0.072) |
| Security workers | -0.065 | 0.226 |
|  | (0.052) | (0.234) |
| Qualified workers in the agricultural industry | 0.049 | 0.044 |
|  | (0.058) | (0.073) |
| Artisans and qualified workers in the industry, building and mining sectors | -0.013 | 0.195 |
|  | (0.041) | (0.126) |
| Industrial machinery and installations´ operators | -0.004 | 0.335** |
|  | (0.020) | (0.119) |
| Unqualified workers | -0.030 | 0.159 |
|  | (0.024) | (0.093) |
| **Gender** |  |  |
| Male | Ref. | |
| Female | 0.038*** | 0.218*** |
|  | (0.012) | (0.035) |
| **Severity** |  |  |
| *Age (years)* |  | |
| 18 to 24 | Ref. | |
| 35 to 44 | -0.047*** | 0.018 |
|  | (0.011) | (0.038) |
| 45 to 64 | -0.039*** | -0.001 |
|  | (0.009) | (0.029) |
| 65 to 75 | -0.065*** | -0.003 |
|  | (0.016) | (0.050) |
| 75 or more | -0.131*** | -0.198*** |
|  | (0.025) | (0.057) |
| **Chronic illness** |  | |
| Presence of chronic illness | 0.053*** | 0.192*** |
|  | (0.013) | (0.035) |
| **Public visits** |  |  |
| 1 visit | Ref. | |
| 2 visits | 0.029 | 0.032 |
|  | (0.017) | (0.028) |
| 3 or more visits | 0.039* | -0.172*** |
|  | (0.019) | (0.040) |
| **Area of residence** |  |  |
| Urban | Ref. | |
| Rural | -0.237*** | -0.031 |
|  | (0.044) | (0.043) |
| **Citizenship status** |  |  |
| Native Spanish | Ref. | |
| Spanish acquired | 0.060*** | -0.166 |
|  | (0.015) | (0.138) |
| Foreign | 0.033 | -0.120* |
|  | (0.030) | (0.067) |
| **Fluency** |  |  |
| High | Ref. | |
| Medium | -0.068** | -0.328 |
|  | (0.029) | (0.598) |
| Low | -0.105 | 0.008 |
|  | (0.071) | (0.139) |
| **Private healthcare visits** |  |  |
| 0 visits | Ref. | |
| 1 to 2 visits | 0.007 | 0.168* |
|  | (0.020) | (0.080) |
| 3 or more visits | 0.128** | 0.017 |
|  | (0.048) | (0.072) |
| **Region fixed effects** | Yes | Yes |
| **Year fixed effects** | Yes | Yes |
| **Specialist FE** | No | Yes |
| **Constant** | 0.957*** | 3.601*** |
|  | (0.039) | (0.084) |
| **Observations** | 24,935 | 6,825 |
| **R-squared** | 0.261 | 0.050 |
| **AIC** | 50905 | 21522 |
| **BIC** | 51035 | 21631 |

^1^ For primary care, our individual of reference is a male with no studies; inactive; between 18 and 34 years old; no chronic disease; one visit to public GP; no visit to private GPs; Spanish; fluent and living in an urban area. For specialist consultations, our individual of reference is a male who has visited Traumatology services; with no studies; employed; between 18 and 34 years old; with no chronic disease; one visit to public specialist services; no visits to private specialist doctor; Spanish; fluent and living in a urban area.

^2^ Full model specification: SES and severity variables + area of residence+ immigrant status + private healthcare utilization + region-year fixed effects.

^3^ Note: *** p<0.01, ** p<0.05, * p<0.1

**Table S3** Quantile estimations for waiting time in specialist services including disaggregated employment status

| **Waiting time for specialist** | **Q(0.10)** | **Q(0.25)** | **Q(0.50)** | **Q(0.75)** | **Q(0.90)** |
| --- | --- | --- | --- | --- | --- |
| **Education** |  |  |  |  |  |
| Primary | -0.0235 | -0.118 | 0.00259 | -0.0367 | 0.0433 |
| Secondary | -0.199 | -0.276** | -0.0962 | -0.0926 | 0.00312 |
| University | -0.134 | -0.218 | -0.136 | -0.11 | -0.0591 |
| **Employment status** |  |  |  |  |  |
| Retirement pensioner | 0.125 | 0.158* | 0.0993 | 0.133** | 0.0689 |
| Unemployed | 0.348*** | 0.221** | 0.0912 | 0.137** | 0.0851 |
| Managers (private and public institutions) | -0.0879 | 0.134 | 0.025 | 0.153 | -0.0642 |
| Technical and profesional scientists and intellectuals | 0.0637 | -0.129 | -0.0618 | -0.00464 | 0.0758 |
| Support technicians and professionals | 0.0958 | 0.000607 | -0.0134 | 0.0384 | 0.083 |
| Office workers | -0.107 | 0.168 | 0.15 | 0.000631 | -0.101 |
| Hospitality and shop workers | 0.0458 | 0.136 | 0.0413 | 0.117 | 0.106 |
| Security and protection workers | -0.0024 | 0.0504 | -0.199 | 0.0314 | 0.335 |
| Qualified workers in the agricultural industry | -0.0468 | 0.168 | 0.113 | 0.182 | -0.0738 |
| Artisans and qualified workers in the industry, building and mining sectors | 0.186 | 0.166 | 0.0655 | 0.12 | 0.142 |
| Industrial machinery and installations´ operators | 0.346 | 0.417*** | 0.199 | 0.145 | 0.293 |
| Unqualified workers | 0.161 | 0.0767 | 0.0886 | 0.227** | 0.125 |
| **Gender** |  |  |  |  |  |
| Female | 0.313** | 0.248*** | 0.180*** | 0.161*** | 0.106** |
| **Age (years)** |  |  |  |  |  |
| 35 to 44 | 0.0995 | 0.0231 | 0.0127 | -0.0442 | -0.125** |
| 45 to 64 | 0.167 | -0.02 | -0.0108 | -0.0571 | -0.0687 |
| 65 to 74 | 0.0258 | 0.000425 | -0.0591 | -0.0538 | -0.0276 |
| 75 or more | -0.0878 | -0.338** | -0.183** | -0.174*** | -0.0722 |
| **Chronic illness** |  |  |  |  |  |
| Presence of chronic illness | 0.126 | 0.180** | 0.206*** | 0.201*** | 0.171*** |
| **Number of visits to GP** |  |  |  |  |  |
| 2 visits | 0.252** | 0.127** | 0.0519 | -0.021 | -0.0833* |
| 3 or more visits | 0.00293 | -0.111** | -0.150*** | -0.240*** | -0.258*** |
| **Area of residence** |  |  |  |  |  |
| Rural | -0.0681 | -0.0643 | -0.0702 | -0.00355 | 0.00946 |
| **Citizenship status** |  |  |  |  |  |
| Spanish | -0.0173 | -0.217 | -0.00882 | -0.13 | -0.169 |
| Foreign | -0.322 | -0.0692 | -0.149 | -0.222* | -0.237** |
| **Fluency** |  |  |  |  |  |
| Medium | -1.591 | -0.882 | -0.0272 | 0.164 | 0.0622 |
| Low | 0.127 | -0.0654 | 0.159 | 0.132 | 0.265 |
| **Private consultations** |  |  |  |  |  |
| 1 or 2 visits | 0.12 | 0.165 | 0.146 | 0.138* | 0.0632 |
| 3 or more visits | -0.393 | 0.0341 | 0.154 | 0.139 | 0.022 |
| **Year fixed effects** | Yes | Yes | Yes | Yes | Yes |
| **Region fixed effects** | Yes | Yes | Yes | Yes | Yes |
| **Specialist fixed effects** | Yes | Yes | Yes | Yes | Yes |
| **Constant** | 1.992*** | 3.109*** | 3.793*** | 4.314*** | 4.977*** |
| **Observations** | 6825 | 6825 | 6825 | 6825 | 6825 |

^1^ Our individual of reference is a male who has visited Traumatology services; with no studies; inactive; between 18 and 34 years old; with no chronic disease; one visit to public specialist services; no visit to a private specialist doctor; Spanish citizenship; fluent and living in a urban area.

^2^ Full model specification: SES and severity variables + area of residence+ immigrant status + private healthcare utilization + region and -year fixed effects.

^3^ Note: *** p<0.01, ** p<0.05, * p<0.1

**Supplementary material 3** Estimations of waiting times for specialist services with the Spanish NHS including household income

**Table S4** OLS estimations for waiting times by SES including disaggregated employment status and household income.

| **Waiting times** | **Primary care** | **Specialist care** |
| --- | --- | --- |
|  |  |  |
| **Education** |  |  |
| No qualifications | Ref. | |
| Primary studies | -0.016 | -0.032 |
|  | (0.026) | (0.063) |
| Secondary studies | -0.007 | -0.081** |
|  | (0.030) | (0.034) |
| University studies | -0.035 | -0.106** |
|  | (0.033) | (0.043) |
| **Employment status** |  |  |
| Inactive | Ref. | |
| Retirement pensioner | -0.019 | 0.159 |
|  | (0.017) | (0.099) |
| Unemployed | -0.020 | 0.165*** |
|  | (0.011) | (0.049) |
| Managers (private and public institutions) | -0.077** | 0.089 |
|  | (0.030) | (0.098) |
| Technical and profesional scientists and intellectuals | -0.075** | 0.129 |
|  | (0.028) | (0.131) |
| Support technicians and professionals | -0.005 | -0.003 |
|  | (0.045) | (0.094) |
| Office workers | 0.010 | 0.189 |
|  | (0.055) | (0.165) |
| Hospitality and shop workers | -0.019 | 0.118 |
|  | (0.029) | (0.076) |
| Security and protection workers | -0.161*** | 0.410 |
|  | (0.047) | (0.262) |
| Qualified workers in the agricultural industry | -0.001 | -0.056 |
|  | (0.070) | (0.129) |
| Artisans and qualified workers in the industry, building and mining sectors | -0.040 | 0.167 |
|  | (0.038) | (0.133) |
| Industrial machinery and installations´ operators | -0.005 | 0.361** |
|  | (0.021) | (0.129) |
| Unqualified workers | -0.029 | 0.153 |
|  | (0.033) | (0.089) |
| **Household income** |  |  |
| Q1 income | Ref. | |
| Q2 income | -0.020 | -0.034 |
|  | (0.015) | (0.029) |
| Q3 income | -0.003 | -0.038 |
|  | (0.020) | (0.043) |
| Q4 income | -0.012 | -0.184** |
|  | (0.018) | (0.075) |
| Q5 income | -0.017 | -0.356 |
|  | (0.028) | (0.208) |
| **Region fixed effects** | Yes | Yes |
| **Year fixed effects** | Yes | Yes |
| **Region-Trend** | Yes | Yes |
| **Specialist fixed effects** | No | Yes |
| **Constant** | 0.995*** | 3.466*** |
|  | (0.050) | (0.076) |
| **Observations** | 16,994 | 5,104 |
| **R-squared** | 0.237 | 0.060 |
| **AIC** | 34885 | 16045 |
| **BIC** | 35008 | 16149 |

^1^ For primary care, our individual of reference is a male with no studies; inactive; Q1 household income (below 600 monthly euros); between 18 and 34 years old; no chronic disease; one visit to public GPno visit to private GPs; Spanish and living in a urban area. For specialist consultations, our individual of reference is a male who has visited Traumatology services; with no studies; Q1 household income (below 600 monthly euros); inactive; between 18 and 34 years old; with no chronic disease; no visits to private specialist doctor; Spanish and living in a urban area.

^2^ Full model specification: SES and severity variables + area of residence+ immigrant status + private healthcare utilization + region-year fixed effects.

^3^ Note: *** p<0.01, ** p<0.05, * p<0.1

**Table S5** Quantile estimations for waiting times by SES including disaggregated employment status and household income.

| **Waiting time for specialist** | **Q(0.10)** | **Q(0.25)** | **Q(0.50)** | **Q(0.75)** | **Q(0.90)** |
| --- | --- | --- | --- | --- | --- |
| **Education** |  |  |  |  |  |
| Primary | -0.123 | -0.132 | 0.0148 | 0.00158 | 0.0728 |
| Secondary | -0.0826 | -0.255** | -0.0529 | -0.0345 | 0.00664 |
| University | 0.018 | -0.167 | -0.119 | -0.161** | -0.112 |
| **Employment status** |  |  |  |  |  |
| Retirement pensioner | 0.12 | 0.146** | 0.0815 | 0.201*** | 0.103** |
| Unemployed | 0.299** | 0.213*** | 0.0866 | 0.176*** | 0.114** |
| Managers (private and public institutions) | 0.0571 | 0.216** | 0.103 | 0.202* | -0.068 |
| Technical and profesional scientists and intellectuals | 0.0682 | -0.121 | 0.0676 | 0.154* | 0.0971 |
| Support technicians and professionals | 0.199 | 0.0658 | -0.00585 | 0.00851 | 0.0925 |
| Office workers | -0.268 | 0.0379 | 0.123 | 0.0753 | 0.0205 |
| Hospitality and shop workers | 0.146 | 0.186 | 0.0774 | 0.127 | 0.064 |
| Securityand protection workers | 0.217 | 0.197 | 0.292 | 0.167 | 0.525 |
| Qualified workers in the agricultural industry | -0.584 | 0.162 | 0.0272 | 0.235** | -0.099 |
| Artisans and qualified workers in the industry, building and mining sectors | 0.122 | 0.103 | -0.00149 | 0.148 | 0.216* |
| Industrial machinery and installations´ operators | 0.373 | 0.431*** | 0.113 | 0.232** | 0.218 |
| Unqualified workers | 0.206 | 0.0969 | 0.0834 | 0.249*** | 0.217*** |
| **Household income** |  |  |  |  |  |
| Q2 | -0.0477 | 0.0302 | -0.0778 | -0.049 | 0.0522 |
| Q3 | -0.0483 | -0.0085 | -0.069 | 0.0419 | 0.122 |
| Q4 | -0.235 | -0.0601 | -0.247** | -0.0953 | 0.0401 |
| Q5 | -0.397 | -0.314** | -0.713** | -0.286** | -0.202 |
| **Gender** |  |  |  |  |  |
| Female | 0.314** | 0.267*** | 0.158*** | 0.205*** | 0.127*** |
| **Age (years)** |  |  |  |  |  |
| 35 to 44 | 0.168 | 0.0903 | 0.0611 | -0.0296 | -0.0816 |
| 45 to 64 | 0.227** | 0.0246 | 0.0274 | -0.0736 | -0.071 |
| 65 to 74 | 0.169 | 0.0704 | 0.0443 | -0.0236 | 0.0478 |
| 75 or more | 0.144 | -0.193* | -0.0682 | -0.124* | -0.0354 |
| **Chronic illness** |  |  |  |  |  |
| Presence of chronic illness | 0.160** | 0.159*** | 0.197*** | 0.197*** | 0.195*** |
| **Number of visits to GP** |  |  |  |  |  |
| 2 visits | 0.247*** | 0.107** | 0.0334 | -0.0189 | -0.0559 |
| 3 or more visits | 0.0544 | -0.0871 | -0.128*** | -0.210*** | -0.205*** |
| **Area of residence** |  |  |  |  |  |
| Rural | 0.00745 | -0.041 | -0.0667 | -0.0188 | 0.0232 |
| **Citizenship status** |  |  |  |  |  |
| Spanish | -0.13 | -0.225 | -0.0146 | -0.187** | -0.0921 |
| Foreign | -0.278 | 0.00257 | -0.169* | -0.170** | -0.223*** |
| **Fluency** |  |  |  |  |  |
| Medium | 0.137 | 0.0287 | 0.240* | 0.166 | 0.2 |
| Low | 0.334 | -0.447 | 0.113 | 0.207 | 0.241 |
| **Private consultations** |  |  |  |  |  |
| 1 or 2 visits | 0.255* | 0.152 | 0.114 | 0.103* | 0.0469 |
| 3 or more visits | -0.629** | 0.0149 | 0.186** | 0.170* | 0.0572 |
| **Year fixed effects** | Yes | Yes | Yes | Yes | Yes |
| **Region fixed effects** | Yes | Yes | Yes | Yes | Yes |
| **Specialist fixed effects** | Yes | Yes | Yes | Yes | Yes |
| **Constant** | 1.793*** | 2.934*** | 3.745*** | 4.128*** | 4.688*** |
| **Observations** | 5104 | 5104 | 5104 | 5104 | 5104 |

^1^ Our individual of reference is a male who has visited Traumatology services; with no studies; Q1 household income (below 600 monthly euros); inactive; between 18 and 34 years old; with no chronic disease; one visit to public specialist care; no visit to a private specialist doctor; Spanish citizenship; fluent and living in a urban area.

^2^ Full model specification: SES and severity variables + area of residence+ immigrant status+fluency + private healthcare utilization + region and -year fixed effects.

^3^ Note: *** p<0.01, ** p<0.05, * p<0.1

**Supplementary material 4** Estimations of waiting times for specialist services with the Spanish NHS including household income

**Table S6** OLS estimations for waiting times in primary care by SES including disaggregated employment status and personal income.

| **Waiting times** | **Primary care** |
| --- | --- |
|  |  |
| **Education** |  |
| No qualifications | Ref. |
| Primary studies | -0.028 |
|  | (0.021) |
| Secondary studies | -0.037 |
|  | (0.025) |
| University studies | -0.047* |
|  | (0.027) |
| **Employment status** |  |
| Inactive | Ref. |
| Retirement pensioner | -0.019 |
|  | (0.017) |
| Unemployed | -0.020 |
|  | (0.011) |
| Managers (private and public institutions) | -0.077** |
|  | (0.030) |
| Technical and profesional scientists and intellectuals | -0.075** |
|  | (0.028) |
| Support technicians and professionals | -0.005 |
|  | (0.045) |
| Office workers | 0.010 |
|  | (0.055) |
| Hospitality and shop workers | -0.019 |
|  | (0.029) |
| Security and protection workers | -0.161*** |
|  | (0.047) |
| Qualified workers in the agricultural industry | -0.001 |
|  | (0.070) |
| Artisans and qualified workers in the industry, building and mining sectors | -0.040 |
|  | (0.038) |
| Industrial machinery and installations´ operators | -0.005 |
|  | (0.021) |
| Unqualified workers | -0.029 |
|  | (0.033) |
| **Personal income** |  |
| Q1 income | Ref. |
| Q2 income | -0.001 |
|  | (0.015) |
| Q3 income | -0.008 |
|  | (0.038) |
| Q4 income | 0.003 |
|  | (0.054) |
| Q5 income | 0.142 |
|  | (0.149) |
| **Region fixed effects** | Yes |
| **Year fixed effects** | Yes |
| **Region-Trend** | Yes |
| **Specialist fixed effects** | No |
| **Constant** | 0.977*** |
|  | (0.044) |
| **Observations** | 14,344 |
| **R-squared** | 0.276 |
| **AIC** | 29998 |
| **BIC** | 30120 |

^1^ Full model: our individual of reference is a male with no studies; inactive; Q1 household income (below 600 monthly euros); between 18 and 34 years old; no chronic disease; one visit to public GPno visit to private GPs; Spanish and living in a urban area.

^2^Note: Standard errors are shown in parentheses. *** p<0.01, ** p<0.05, * p<0.1

^3^ Akaike Information Criterion (AIC) and the Bayesian Information Criterion (BIC) were calculated to compare the goodness-of-the-fit. A smaller AIC or BIC indicates a better-fitting model.

*^4^*Source: *The authors, based on the Spanish National Health Barometer*

**Supplementary material 5** Differences in waiting times by region.

**Figure 1.** Coefficients for regional fixed effects. Waiting times for primary care (2011-2019).


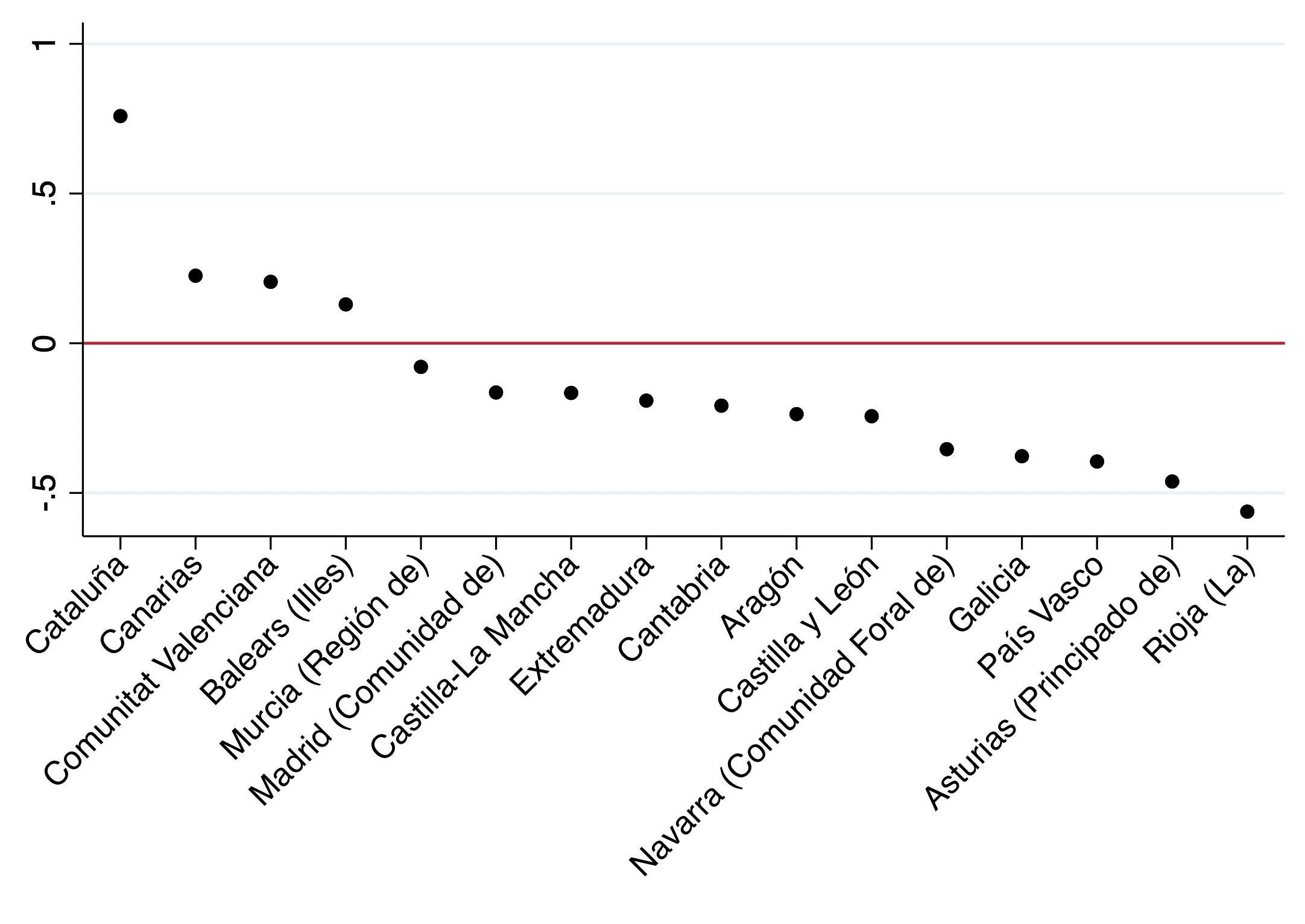


Notes:

1. Andalucía is the region of reference.
2. Coefficients from OLS estimations ranked from highest to lowest (Table 2).

**Figure S2**. Coefficients for regional fixed effects .Waiting times for specialist service (2011-2013).


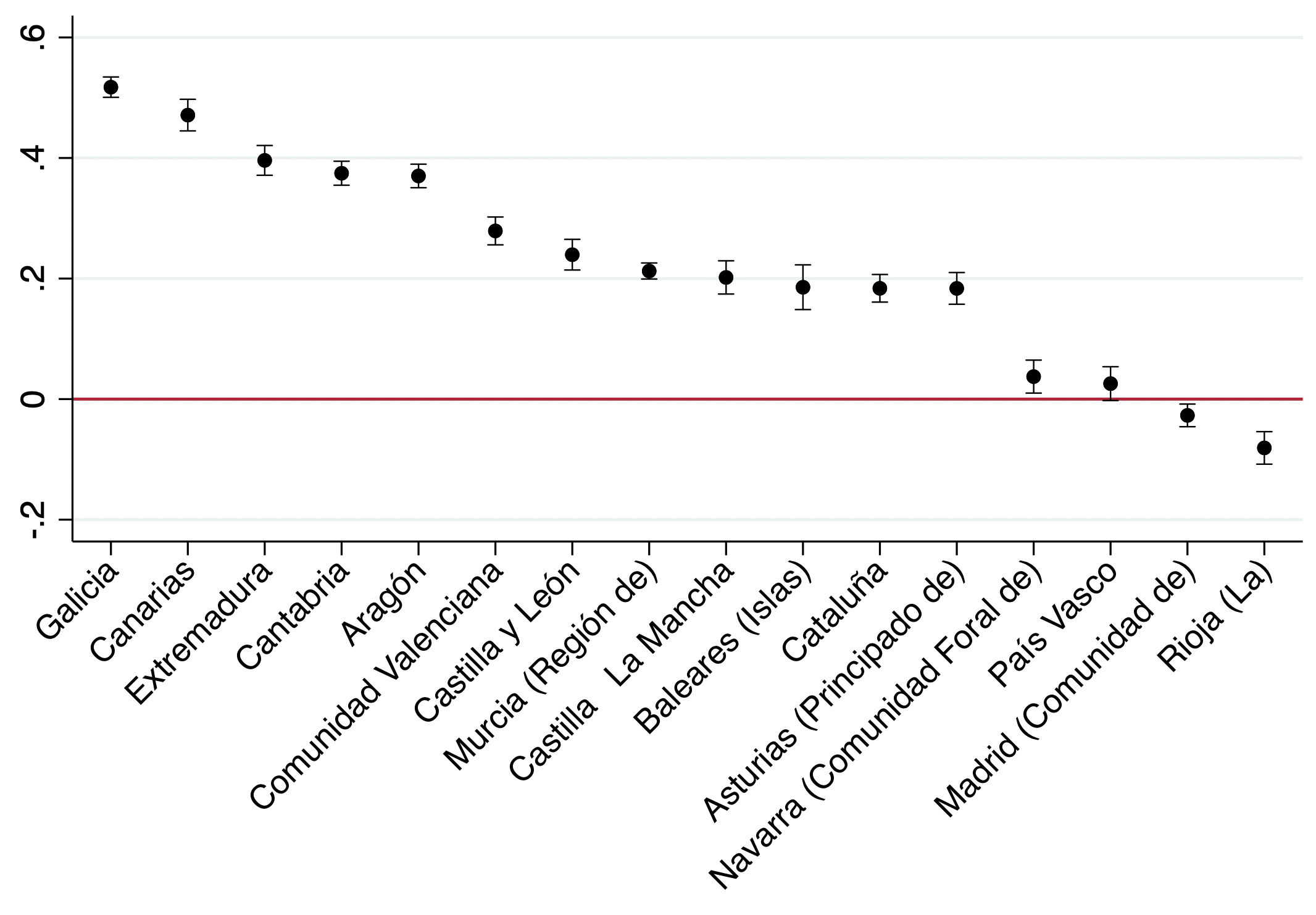


Notes:

1. Andalucía is the region of reference.
2. Coefficients from OLS estimations ranked from highest to lowest (Table 3).
